# Computational design of a cyclic peptide that inhibits the CTLA4 immune checkpoint†

**DOI:** 10.1039/d2md00409g

**Published:** 2023-03-01

**Authors:** Ravindra Thakkar, Deepa Upreti, Susumu Ishiguro, Masaaki Tamura, Jeffrey Comer

**Affiliations:** a Department of Anatomy and Physiology, Kansas State University 1620 Denison Avenue Manhattan Kansas USA jeffcomer@ksu.edu +1 785 532 6311

## Abstract

Proteins involved in immune checkpoint pathways, such as CTLA4, PD1, and PD-L1, have become important targets for cancer immunotherapy; however, development of small molecule drugs targeting these pathways has proven difficult due to the nature of their protein–protein interfaces. Here, using a hierarchy of computational techniques, we design a cyclic peptide that binds CTLA4 and follow this with experimental verification of binding and biological activity, using bio-layer interferometry, cell culture, and a mouse tumor model. Beginning from a template excised from the X-ray structure of the CTLA4:B7-2 complex, we generate several peptide sequences using flexible docking and modeling steps. These peptides are cyclized head-to-tail to improve structural and proteolytic stability and screened using molecular dynamics simulation and MM-GBSA calculation. The standard binding free energies for shortlisted peptides are then calculated in explicit-solvent simulation using a rigorous multistep technique. The most promising peptide, cyc(EIDTVLTPTGWVAKRYS), yields the standard free energy −6.6 ± 3.5 kcal mol^−1^, which corresponds to a dissociation constant of ∼15 μmol L^−1^. The binding affinity of this peptide for CTLA4 is measured experimentally (31 ± 4 μmol L^−1^) using bio-layer interferometry. Treatment with this peptide inhibited tumor growth in a co-culture of Lewis lung carcinoma (LLC) cells and antigen primed T cells, as well as in mice with an orthotropic Lewis lung carcinoma allograft model.

## Abstract 2

A cyclic peptide targeted to the immune checkpoint protein CTLA4 is designed using a hierarchy of computational methods, demonstrated to bind by a physical measurement, and shown to inhibit tumor growth in cell culture and in mice.

## Graphical abstract

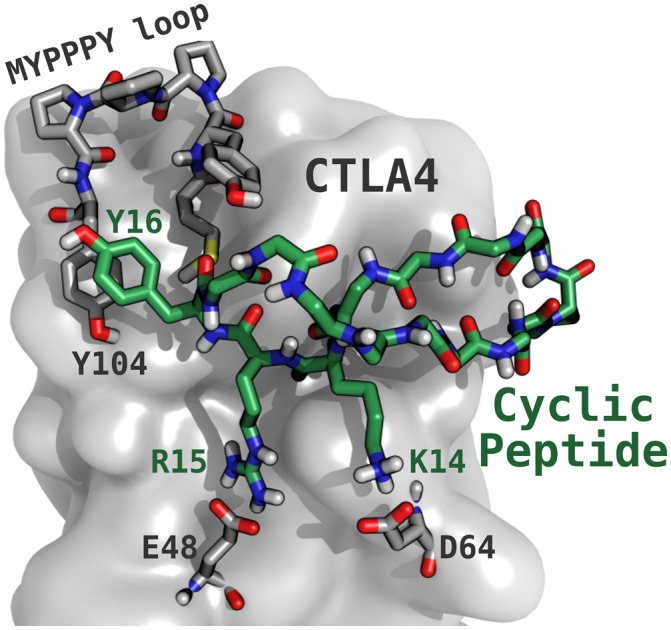

## Introduction

Immune cells typically infiltrate the tumor microenvironment and have the ability to detect cancer-specific antigens; however, cancer cells often evolve the ability to hijack self-tolerance mechanisms, inhibiting the immune response and allowing them to evade destruction.^1,2^ These self-tolerance mechanisms are referred as immune checkpoints and involve inhibitory receptors such as CTLA4, PD1, TIM3, LAG3, and BTLA, which are expressed on immune cells. Immunotherapies based on antibodies that bind these receptors (CTLA4, PD1) or their natural ligands (PD-L1 for the PD1/PD-L1 pathway) have dramatically improved clinical results for some cancers. The first putative immune checkpoint inhibitor approved by the US Food and Drug Administration was ipilimumab,^3^ a monoclonal antibody that binds to CTLA4, and is thought to work by preventing binding of its natural ligands B7-1 and B7-2 (also known as CD80 and CD86).^2,4–6^ However, the precise mechanism of ipilimumab may be more complex than first thought.^7^ At the current time, approved immune checkpoint inhibitors that target PD1 and PD-L1 also find clinical use. Targeting both the CTLA4 and PD1 pathways appears promising and combined therapies can yield improved clinical outcomes for some cancers.^8^

**Fig. 1:**
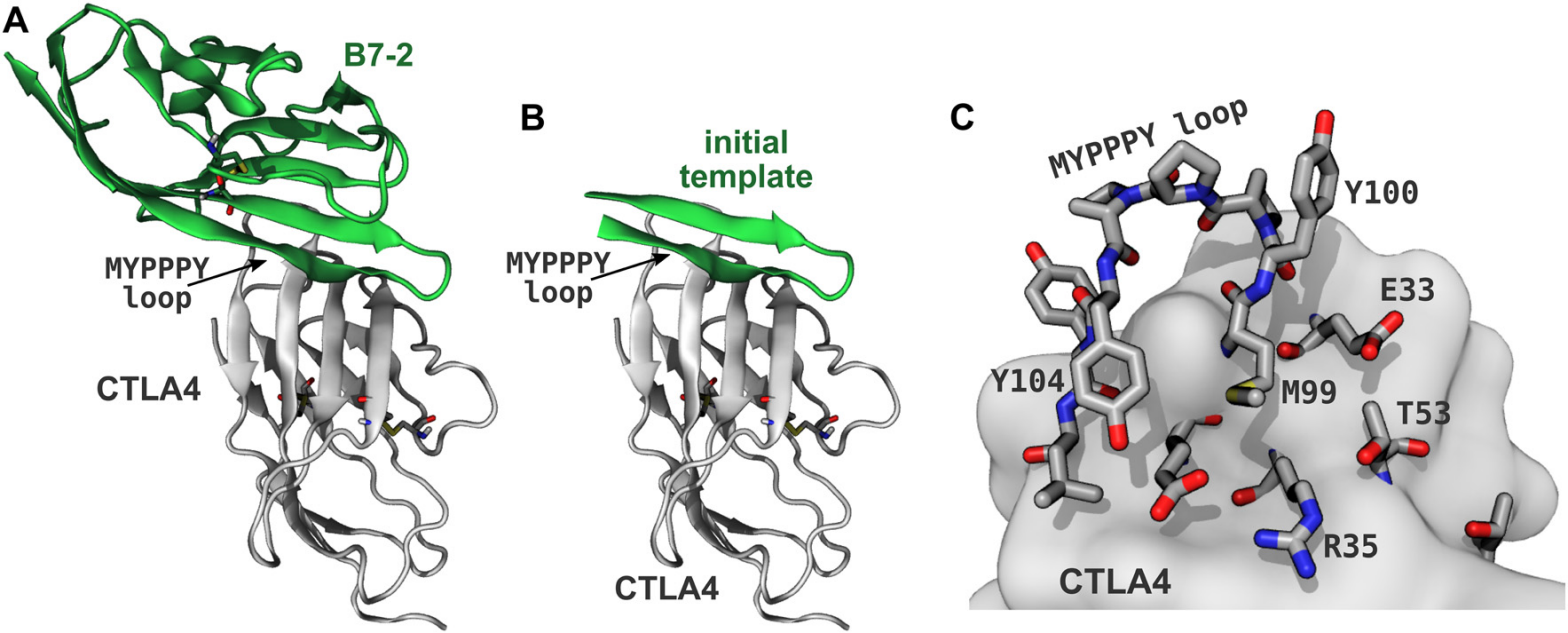
Generating the template peptide. (A) CTLA4 (gray) bound to its ligand B7-2 (green) from a published X-ray structure (PDB ID: 1I85).^15^ Disulfide bridges are shown explicitly. (B) A template for the designed peptides created by extracting residues 85–101 of B7-2. (C) Binding site of CTLA4. Residues within 5 Å of the B7-2 ligand in the X-ray structure are explicitly shown.

Thus far, all successful immune checkpoint inhibitors have been monoclonal antibodies. However, such antibodies are difficult and expensive to produce and store,^9^ leading to unfavorable cost effectiveness in some cases.^10^ Small-molecule immune checkpoint inhibitors could provide simpler synthesis and lower costs, but developing potent small molecules has proven difficult due to the relatively flat protein–protein interfaces of immune checkpoint receptors and many of their ligands.^9,11,12^ Most proteins involved in inhibitory immune checkpoint signaling, including CTLA4, B7-1, B7-2, PD1, PD-L1, PD-L2, TIM3, LAG3, BTLA, B7-H4 (VTCN1), and B7-H3 (CD276), have immunoglobulin-like V (IgV) domains and some also possess similar immunoglobulin-like C1 or C2 domains.^13^ These domains have a β-sandwich structure,^14^ consisting of two β-sheets lying face-to-face, which are tethered together by one or more disulfide bridges. The binding interfaces of these domains are typically found on the relatively flat surfaces of these β-sheets, which include no obviously druggable pockets. For instance, the human CTLA4:B7-2 complex,^15^ shown in Fig. 1, has a binding interface involving the β-sheet faces of CTLA4 and B7-2, as well as the conserved MYPPPY loop^16,17^ of CTLA4 (residues 99–104, using the residue numbering of PDB ID 1I85). Although this paper focuses on designing peptides that bind to this interface of CTLA4, we expect that the design principles might be used to design inhibitors for similar proteins involved immune checkpoint signaling.

While protein–protein interfaces can be difficult targets for conventional small molecule drugs, peptides, by their nature, should be able to mimic natural protein–protein interactions.^18^ Moreover, the facile synthesis and modification of peptides make them a promising alternative to antibodies and other drugs.^19^ Various studies have reported the therapeutic applications of synthetic peptides in drug delivery, cell membrane penetration, specific cell targeting and activation of immune response.^20,21^ Tens of therapeutic peptides are already approved to treat cancer, diabetes, and cardio-vascular diseases.^22^ One major challenge to the use of therapeutic peptides is rapid degradation by proteolytic enzymes present in the physiological environment.^18^ However, this challenge can be overcome by taking inspiration from natural protease-resistant peptides, which are often cyclized to improve the proteolytic stability.^23,24^ Like natural peptides, synthetic peptides can be cyclized by one or more disulfide bridges or by peptide linkages involving the N-terminus and C-terminus, or side chains (usually those of Lys, Asp, or Glu).^23,25^ Furthermore, crosslinks of various chemistries (referred to as “staples”) can be used to cyclize peptides in ways never found in nature.^18,24^ Cyclization also helps to stabilize the secondary structure of peptides^26–28^ and can also improve binding affinity by reducing the entropy of the unbound state and therefore the entropy lost upon binding.^12,29,30^

Computational protein design tools have matured over the last decade, enabling rational design of peptides with desired structure and function.^31^ Molecular simulation has been used to assist in the design of a disulfide cyclized peptide that binds to PD1 and likely affects the PD1/PD-L1 checkpoint pathway.^32^ More recently, Bryan *et al.*^33^ used the protein modeling program Rosetta to design a structured mini-protein that binds to PD1 and acts as an agonist, in contrast to approved PD1-binding antibodies, which are antagonists. Combining Rosetta with molecular dynamics simulation and free-energy calculation techniques has much promise for efficient peptide design.^34^ In this paper, we combine Rosetta, molecular dynamics, and a rigorous multistep method to compute the binding free energy^35,36^ to design a CTLA4-binding peptide. We then verify its affinity experimentally using the bio-layer interferometry (BLI) method^37^ and determine its biological effect on the CTLA4 checkpoint pathway using cell culture experiments and mouse models.

## Methods

### Template structure

An X-ray structure of the CTLA4:B7-2 protein complex (Fig. 1A) was obtained from Protein Data Bank (PDB ID: 1I85).^15^ The CTLA4 structure was missing some residues (residues 27–30 and 42–44, inclusive), which were inserted by CHARMM-GUI,^38^ using the GalaxyFill algorithm.^39^ The hotspot residues at the binding interface between the CTLA4 and B7-2 proteins were predicted using the KFC web server.^40,41^ The binding site for B7-2 on CTLA4 is shown in Fig. 1C, highlighting residues having atoms within 5 Å of any atoms of B7-2 in the X-ray structure A fragment of the B7-2 protein comprising residue numbers 85 to 101 inclusive, which was found to be in close contact with CTLA4, was selected as a template for the peptide design (Fig. 1B).

### Peptide design workflow

**Fig. 2:**
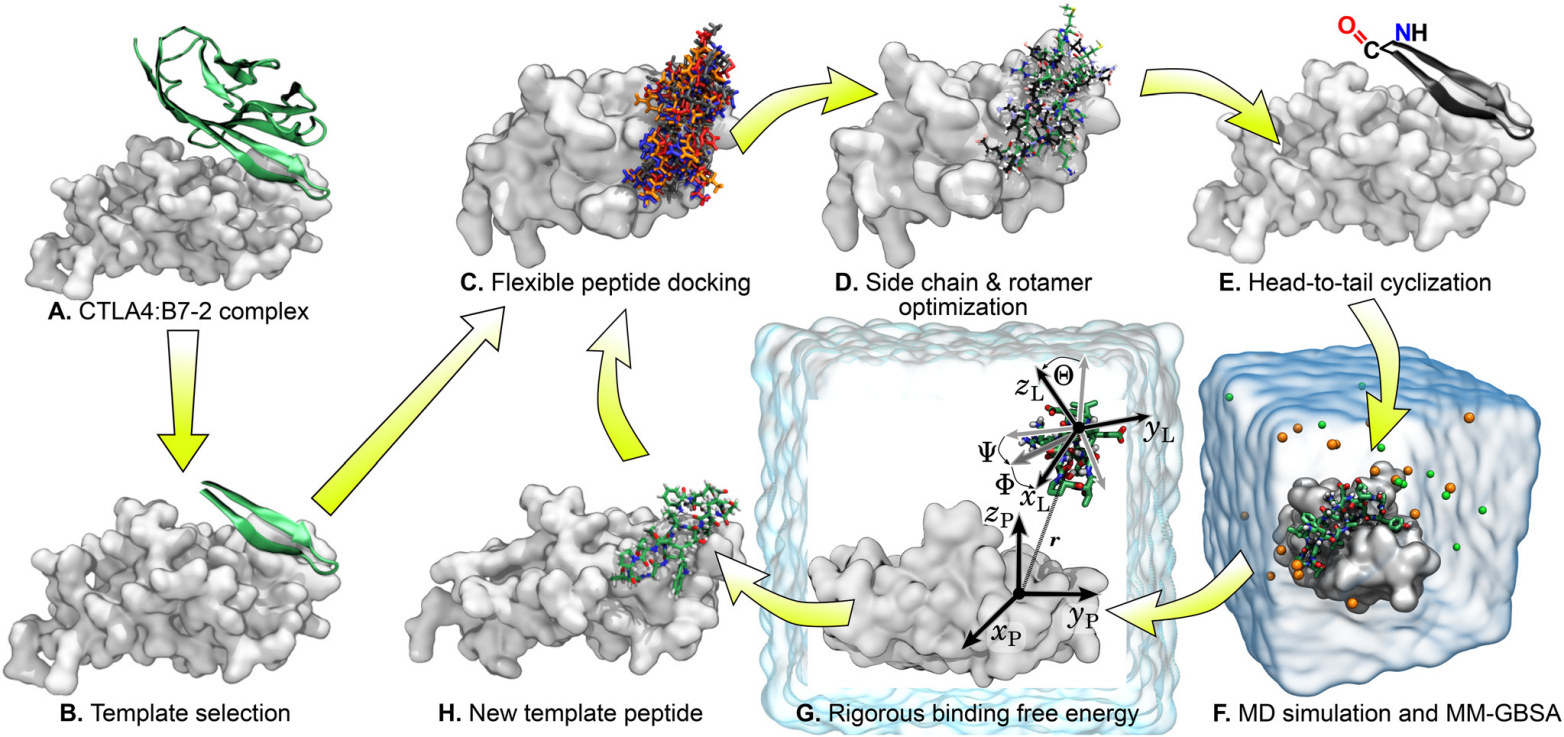
Graphical representation of the workflow for the cyclic peptide design.

Fig. 2 illustrates the workflow for the cyclic peptide design. After selection of the template peptide (Fig. 2A and B), probable poses of the template peptide on the receptor protein CTLA4 were predicted using FlexPepDock *ab initio*^42,43^ (Fig. 2C). FlexPepDock is a module of Rosetta, a protein modeling suite.^44^ Unfortunately, FlexPepDock did not support head-to-tail cyclic peptides, so docking was performed with acyclic peptides. However, the initial β-sheet structure was maintained during docking, keeping the termini in close proximity and minimizing the disruption caused by later linking the N- and C-termini. The secondary structure of the template peptide sequence was predicted using the protein structure prediction web-server Phyre2 (ref. 45) and used as input for FlexPepDock. Hundreds of different poses of the template peptide near the binding site of CTLA4 were generated. A few best-scoring poses with the lowest energies and RMSD values relative to the original template structure were selected. Selected structures were processed with PyRosetta,^46^ a Python-based interface for Rosetta,^44,47^ to optimize side-chain and amino acid sequence under fixed backbone constraints (Fig. 2D). The optimized peptides were cyclized by head-to-tail (N-terminus to C-terminus) peptide bond (Fig. 2E). Molecular dynamics (MD) simulations were carried out to evaluate the stability of their complexes with CTLA4 in explicit water and estimate the binding free energy using the molecular mechanics generalized Born surface area (MM-GBSA) method^48–50^ (Fig. 2F). The MD and MM-GBSA protocols are described in the “Molecular dynamics” and “MM-GBSA free energy estimation” sections below. Some peptides exhibiting longest time bound to CTLA4 or lowest MM-GBSA free energy were subjected to rigorous free energy calculations in explicit solvent MD^36,51,52^ (Fig. 2G), as detailed in the section “Rigorous binding free energy calculations”. Finally, the resulting apparently stable CTLA4:peptide complexes were extracted from the MD simulations and used as new templates for another round of the optimization cycle (Fig. 2H). The sequences of the selected peptides produced by workflow and associated data are given in Table 1. We performed four complete iterations (denoted “design cycles” in Table 1) of the cycle shown in Fig. 2C–H. including the original B7-2 fragment. A cyclized version of the original template with its X-ray coordinates is included and denoted T0, while results using coordinates generated by docking the same peptide are denoted T1 and T2 (with RMSDs of 3.3 and 3.9 Å from the X-ray coordinates).

**Table 1:** Evaluation of the binding affinity for candidate peptides. For each sequence, we include the Rosetta score (lower is more favorable), the time that the peptide remained bound to CTLA4 in an MD simulation, the binding free energy as estimated by the MM-GBSA method, and the binding free energy as estimated by the rigorous explicit-solvent MD method. “Design cycles” refers to the number of times we passed through the design loop shown in Fig. 2 to produce that sequence. T0 is a cyclized version of the template (residues 85–101 of B7-2) with the original X-ray coordinates, and T1 and T2 are redocked coordinates for the same peptide

ID	Sequence	Design cycles	Rosetta score	Time bound (ns)	Δ*G*_MM-GBSA_ (kcal mol^−1^)	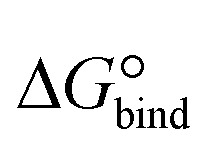 (kcal mol^−1^)
T0	cyc-CIIHHKKPTGMIRIHQM	0	172.4	61.3	−12.6 ± 0.3	
T1	cyc-CIIHHKKPTGMIRIHQM	0	173.6	58.3	−16.4 ± 0.5	
T2	cyc-CIIHHKKPTGMIRIHQM	0	170.8	90.8	−12.2 ± 0.2	
1	cyc-ECRYEPRPEGNILVSYS	1	170.6	660.2	−22.7 ± 0.2	
2	cyc-SIVTKLTPTGWVAASYS	1	175.1	899.4	−26.4 ± 0.1	
3	cyc-KVEFKRTPSGTITVSME	1	165.8	12.8	−10.4 ± 0.6	
4	cyc-KVVYEPKPEGNIVVEYE	1	194.3	48.4	−12.4 ± 0.3	
5	cyc-SAKFEPRPEGNIVVSYG	2	200.6	134.0	−22.7 ± 0.2	
6	cyc-EARYQPRPDGNVLVSYG	2	206.0	245.0	−14.2 ± 0.2	
7	cyc-SAKWNPKPEGAELIEEG	2	222.8	16.0	−7.7 ± 0.7	
8	cyc-SAEFIPTPDGNLLKSSG	2	214.0	13.8	−8.8 ± 0.7	
9	cyc-SIVVVLTPTGWVAASYS	2	157.6	278.0	−26.9 ± 0.3	
10	cyc-EIITKLTPTGWVAASYS	2	157.6	86.2	−19.3 ± 0.4	
11	cyc-SIEMELTPTGWVNKSSS	2	157.9	44.2	−11.3 ± 0.3	
12	cyc-SIITVLTPTGWVAAEFS	2	155.7	1110.6	−32.8 ± 0.1	−10.2 ± 2.4
13	cyc-DIITILTPTGYVAAAYS	3	154.0	395.6	−19.0 ± 0.2	
14	cyc-SIITVLTPTGWVAAYYS	3	155.3	1463.2	−23.3 ± 0.1	
15	cyc-SIQCVLTPTGWVAARYS	3	155.1	42.4	−20.3 ± 0.7	
16	cyc-EIDTVLTPTGWVAKRYS	3	153.8	2000.0^a^	−22.3 ± 0.1	−6.6 ± 3.5
17	cyc-SIRMELTPTGWVAAEYE	3	165.2	119.4	-18.5 ± 0.4	

a Peptide remained bound for the full duration of the simulation (2 μs).

Before using head-to-tail cyclization, we initially attempted to design peptides cyclized by disulfide bridges between terminal cysteines. Table S1† shows data similar to Table 1 for these disulfide cyclized peptides. However, we did not find the results satisfactory, which prompted the move to head-to-tail cyclization. A major contributor to this is the fact that cysteine disulfide bridges do not have an optimal geometry for β-sheets (the C_α_–C_α_ distance is a bit too short). For the disulfide cyclized peptides, the longest time bound was 580 ns and the lowest Δ*G*_MM-GBSA_ was −19.6 ± 0.5 kcal mol^−1^, which compares unfavorably with the corresponding values of >2000 ns and −32.8 ± 0.1 kcal mol^−1^ for the head-to-tail cyclized peptides.

### Molecular dynamics

**Fig. 3:**
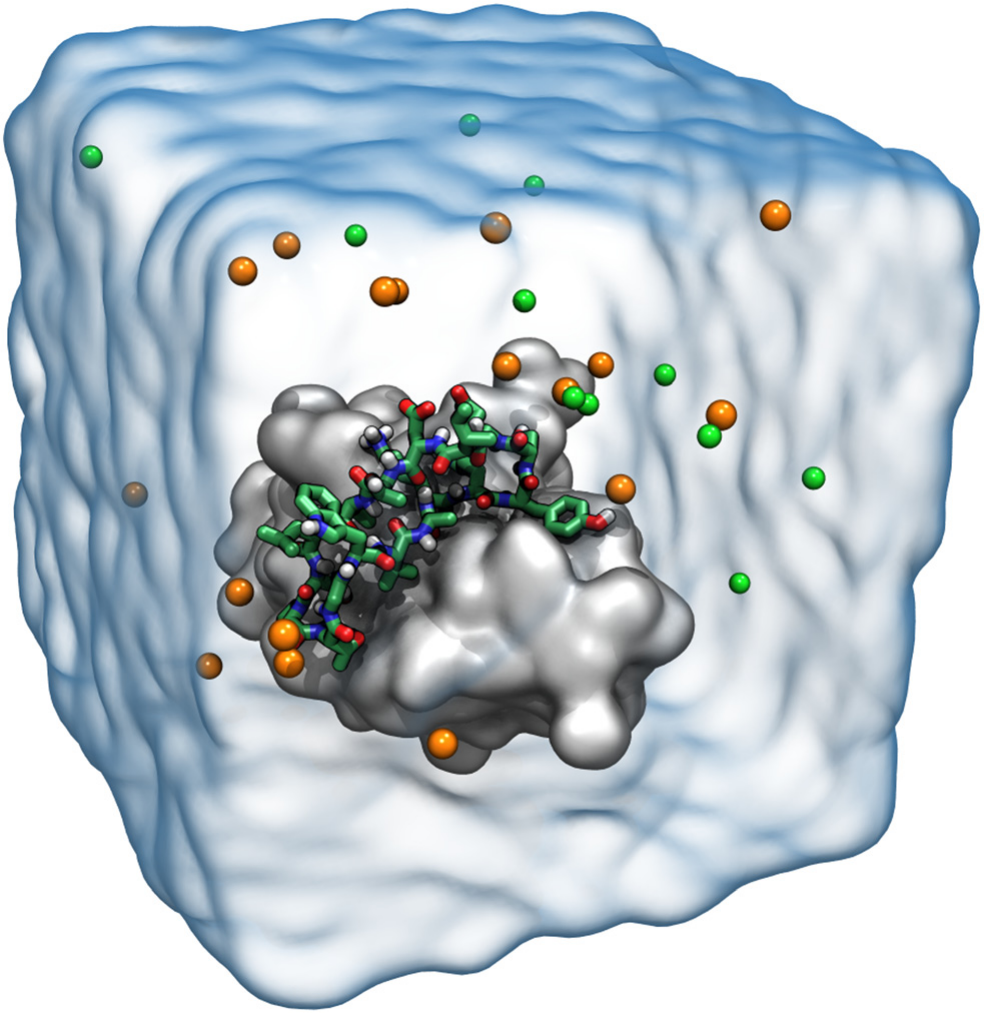
Explicit solvent model of the biomolecular system consisting of the CTLA4 receptor protein, represented as a gray surface, and a designed peptide, represented by bonds (H, white; C, green; N, blue; O, red). Na^+^ and Cl^−^ ions are shown as orange and bright green spheres, respectively. For clarity, the explicit water molecules are shown only as a transparent surface.

Molecular dynamics simulations for each designed peptide bound to the CTLA4 receptor protein were carried out using NAMD 2.13, a scalable molecular dynamics simulation program.^53^ For efficiency, long simulations of peptide 16 bound to CTLA4 (Fig. 6) were performed using NAMD 3alpha6 and the CUDASOAIntegrate option.^54^ The CHARMM36m force field for proteins^55^ was used to define the forces between the atoms. The two terminal residues of resulting peptides were patched by a *trans* head-to-tail (N-terminal to C-terminal) peptide bond. The structures of the complexes were solvated using the CHARMM version of the TIP3P^56^ water model. Salt ions were added to give Na^+^ and Cl^−^ concentrations of approximately 150 mmol L^−1^. Four additional Na^+^ ions were included to neutralize the system. An exemplary simulation system is shown in Fig. 3. VMD was used to aid structure building, analysis, and visualization.^57^

The mass of solute hydrogen atoms was repartitioned to allow a 4 femtosecond timestep to improve computational efficiency.^58^ The Lennard-Jones interactions between pair of atoms was calculated using cutoff distance of 12 Å, smoothly truncated beginning at 10 Å. Water molecules were kept rigid using the SETTLE algorithm^59^ while the length of other covalent bonds involving hydrogen were constrained with RATTLE.^60^ A temperature of 300 K and a pressure of 1.01325 bar were maintained using the Langevin thermostat^61^ and Langevin piston method,^62^ respectively. The electrostatic interactions were calculated by particle mesh Ewald (PME) method using grid spacing of 1.2 Å.^63^ Energy minimization of 2000 steps was performed for each system.^64^ After minimization, 0.1 ns of molecular dynamics were performed with restraints applied to non-hydrogen atoms of the protein, followed by 1.0 ns with restraints applied to the only the C_α_ carbon atoms. The production simulations to screen different sequences were performed without using any restraints and set to stop when the C_α_ atoms of the candidate peptide showed a root mean square displacement (RMSD) of more than 25 Å from their initial positions. The Colvars module of NAMD was used to implement the stopping criterion.^65^ Every 200 ps, a frame of the trajectory was written for analysis.

### MM-GBSA free energy estimation

Subsequently, for each frame of the MD trajectories, the binding free energy of the candidate peptide to the CTLA4 was estimated using the molecular mechanics generalized Born surface area (MM-GBSA) method.^48–50^ To implement the GBSA calculation, the structures of the receptor protein, candidate peptide, and protein : peptide complex were extracted from the output trajectories and snapshots were created. For each snapshot of the receptor protein, candidate peptide, and protein : peptide complex MM-GBSA calculated as Δ*G*_MM-GBSA_ = Δ*G*^protein : peptide^_MM-GBSA_ − Δ*G*^protein^_MM-GBSA_ − Δ*G*^peptide^_MM-GBSA_. The Δ*G* values were calculated using the NAMD implementation of generalized Born implicit solvent with a dielectric constant of 78.5 and a surface tension 0.00542 kcal mol^−1^ Å^−2^. The conformational entropy was not calculated since these calculations were intended for rapid screening and including conformational entropy estimates in the MM-GBSA framework does not necessarily improve agreement with experiment.^66^

### Rigorous binding free energy calculations

The designed cyclic peptides with more favorable MM-GBSA binding affinities for the CTLA4 receptor protein were shortlisted for the rigorous calculation of absolute binding free energy in explicit solvent using the geometric route.^36,51^ The simulation frame having the minimum MM-GBSA energy was selected as the starting structure for the complex and used as input for the binding free energy estimator (BFEE), a plugin of VMD.^52^ To make the free energy calculation feasible, the calculation was partitioned into separate stages (Table 2). The basic idea^35,36,51^ is to calculate the free energy of binding of a conformationally and orientationally restrained ligand (peptide) to the receptor (CTLA4) and then remove the bias of the restraints by calculating the free energy to apply and release these restraints. Conceptually, the process starts by applying a cumulative series of conformational (stage 1) and orientational (stage 2) restraints to the free ligand. The free energy for binding this restrained ligand to the receptor is then calculated in stage 3. Finally, the free energy associated with cumulatively turning off the directional, orientational, and conformational restraints on the receptor-bound ligand are computed (stages 4–9). Note that, for conceptual clarity, we have numbered the stages in the reverse sequence given by the BFEE plugin.^52^

**Table 2:** Free-energy values and simulation times for each stage of the rigorous calculation of the absolute free energy for binding of peptide 16 to CTLA4. Note that the numbering of the stages is reversed to compared that presented in Fu *et al.*^36^ First, we calculate free energies required to apply conformational (stage 1) and orientational restraints (stage 2) to the free peptide. Next, the binding free energy for the complex is calculated with these restraints applied to the peptide, along with directional restraints for the position of the peptide relative to the protein. Finally, we calculate the free energy of releasing these directional, orientational, and conformational restraints. Stage 2 was computed analytically and required no simulation. Stage 3 was calculated using replica-exchange umbrella sampling. All remaining stages were computed with eABF

Stage	System	Action	Free-energy term	Free energy (kcal mol^−1^)	Time (ns)
1	Ligand	Apply conform. restraint	Δ*G*^apply^_RMSD_	+11.97 ± 2.96	389
2	Ligand	Apply orient. restraints	Δ*G*^apply^_*ΘΦΨ*_	+6.61 ± 0.00	0
3	Complex	Binding of restrained ligand	−*k*_B_*T* ln(S*I*C°)	−12.92 ± 0.79	6956
4	Complex	Release *ϕ* direct. restraint	Δ*G*^release^_*ϕ*_	−0.49 ± 0.11	17
5	Complex	Release *θ* direct. restraint	Δ*G*^release^_*θ*_	−0.05 ± 0.01	20
6	Complex	Release *Ψ* orient. restraint	Δ*G*^release^_*Ψ*_	−0.20 ± 0.02	19
7	Complex	Release *Φ* orient. restraint	Δ*G*^release^_*Φ*_	−0.29 ± 0.17	17
8	Complex	Release *Θ* orient. restraint	Δ*G*^release^_*Θ*_	−0.23 ± 0.02	19
9	Complex	Release conform. restraint	Δ*G*^release^_RMSD_	−11.03 ± 1.59	91
Total	—	Sum	Δ*G*°	−6.63 ± 3.45	7528

Because the initial and final states in these stages represent an unrestrained complex and an unrestrained free ligand/protein, the sum of the free energy over all stages is the unbiased binding free energy. The free energy of applying (upper sign) or releasing (lower sign) each restraint is calculated by1
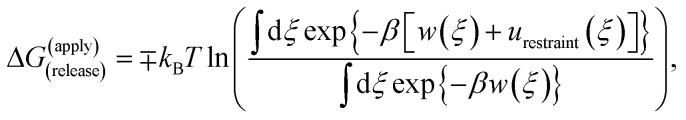
where *β* = 1/*k*_B_*T* is the inverse temperature, *ξ* is the coordinate along which the restraint is applied, *u*_restraint_(*ξ*) is the restraint potential energy, and *w*(*ξ*) is potential of mean force along *ξ* in the absence of this restraint, but in the presence of any restraints previously applied to other coordinates. The standard binding free energy is then calculated by,2
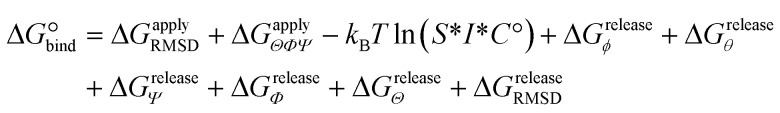
Here, *C*° = 1/1660.539 Å^−3^ is the standard 1 mol L^−1^ concentration and3
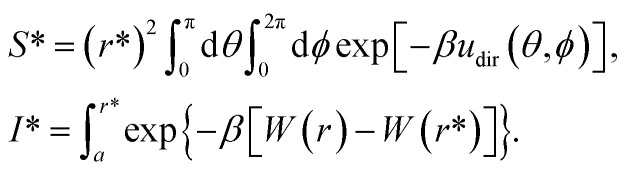
The reference distance r* is 26 Å, *W*(*r*) is the potential of mean force calculated in stage 3, the lower limit of the integral *a* = 16 Å, and *u*_dir_(*θ*, *ϕ*) is the harmonic restraining potential on polar direction angles *θ* and *ϕ*.

Free energies for stages 1 and 4–9 were computed with the extended adaptive biasing force (eABF) method using the defaults of the BFEE plugin,^67–69^ while stage 3 was computed with replica-exchange umbrella sampling^70^ as described in the following paragraph. Stage 2 was computed analytically.^52^ For all stages (except stage 2, which is computed exactly), we estimated the uncertainty by partitioning the adaptive biasing force gradients or umbrella sampling trajectory data into two segments, the first and second halves of each simulation,^71^ and taking the uncertainty to be the maximum absolute difference between the results calculated for each half and the results calculated from the full data set. The potentials of mean force estimated from each of the two segments are shown in Fig. S1 of the ESI† and indicate that the calculations are reasonably well-converged.

### Replica-exchange umbrella sampling

Initially, stage 3 of the rigorous binding free energy calculation was computed by eABF, as in the other stages, using two replicates. However, in both cases, we found that the restrained peptide dissociated from CTLA4 early in the simulation and never again reassociated, leading to poor sampling for *r* < 19 Å. Therefore, to improve sampling over the entire domain, we opted to use the replica-exchange umbrella sampling method.^70^ The radial transition coordinate (*r*) was stratified into 20 windows with centers 16.5 ≤ *r* ≤ 26.0 Å (grid spacing of 0.5 Å). This domain was chosen since the eABF calculation showed a free energy minimum at 17.5 ± 0.2 Å and a plateau for *r* > 25 Å, which was likely reliable since the eABF calculation was well-sampled in the latter region. For each window, the radial separation *r* was restrained to the window center by a harmonic restraint with a spring (force) constant of 10 kcal mol Å^−2^, yielding good overlap of the *r* distribution for adjacent windows (Fig. S2 of the ESI†). Exchanges of the atomic configuration between adjacent windows were attempted every 25 000 steps (0.1 ns of simulated time) and alternated between *w* even or *w* odd for exchanges between *w* and *w* + 1. Acceptance rates between pairs of windows ranged from 8.4% (window 7 ↔ 8) to 30.3% (window 0 ↔ 1). The potentials of mean force were calculated by the weighted histogram analysis method.^72,73^

### Binding assay by bio-layer interferometry

We contracted commercial services (GenScript USA Inc., Piscataway, NJ, USA and LifeTein LLC, Somerset, NJ, USA) to synthesize the cyclic peptide (peptide 16). We also obtained human recombinant CTLA4 (biotinylated) and B7-2 (as positive control) from commercial sources (R&D Systems, Inc., Minneapolis, MN, USA). High precision streptavidin (SAX) BLItz biosensor tips were purchased from FortéBio (Freemont, CA, USA). The binding affinity of peptide 16 for the CTLA4 protein was evaluated using the BLItz bio-layer interferometer (FortéBio, Freemont, CA, USA) at room temperature. The synthesized peptides and proteins (B7-2, the positive control, and biotinylated CTLA4) were stored in powder form at −20 °C after receipt until the experiment. The peptide and proteins were separately dissolved in PBS buffer. The BLItz biosensor tips (FortéBio, Freemont, CA, USA) were used to immobilize the CTLA4 protein and all the tips were hydrated for 15–30 minutes in PBS buffer before each test. A constant signal at the washing step (after loading the CTLA4 on biosensor tips) indicated a immobilization of the CTLA4 protein on the biosensor tip. The PBS buffer without the designed cyclic peptide or any protein was used to record the baseline. A 400 nmol L^−1^ solution of the B7-2 protein, a natural binding partner of CTLA4 was used as positive control and different molar concentrations (150 175 and 200 μmol L^−1^) of the designed peptide were used as the test analyte. The values of association and dissociation constant were obtained using BLItz Pro 1.2 software.

### Cell culture and mouse experiments

The designed peptide was obtained by commercial custom synthesis as described in the previous paragraph. Cell lines for mouse immature dendritic cells (JAWSII, CRL-11904) and mouse Lewis lung carcinoma cells (LLC, CRL-1642) were obtained from American Type Culture Collection (ATCC, Manassas, VA). Cell culture media RPMI 1640, fetal bovine serum, 2-mercaptoethanol, and penicillin–streptomycin solution were purchased from Mediatech, Inc. (Manassas, VA), Biowest (Riverside, MO), Sigma-Aldrich (St. Louis, MO) and Lonza Rockland, Inc. (Allendale, NJ), respectively. The sodium pyruvate (200 mmol L^−1^), l-glutamine (200 mmol L^−1^), 50× Gibco brand antibiotic–antimycotic, 100× MEM non-essential and essential amino acids were obtained from Thermo Fisher Scientific (Waltham, MA). The Ultra-LEAF Purified anti-mouse CD274 (B7-H1, PD-L1) antibody (10F.9G2), the LIVE/DEAD Fixable Violet Dead Cell Stain Kit (Invitrogen) and fluorescent conjugated antibodies targeting FoxP3 (R16-715) were purchased from BioLegend (San Diego, CA), Thermo Fisher Scientific, and BD Bioscience (Franklin Lakes, NJ), respectively. Fluorescently conjugated antibodies targeting mouse CD4 (H129.19), CD8b (YTS156.7.7), IFNγ (XMG1.2), CTLA4 (UC10-4B9), PD-1 (RMP1-30) and isotype controls were obtained from BioLegend. C57BL/6 mice were purchased from Charles River Laboratories International, Inc.

All animal procedures were performed in accordance with the Institutional Animal Care and Use Committee (IACUC) Guidelines of Kansas State University and approved by the Kansas State University IACUC (protocol #4742) and Institutional Biosafety Committee (protocol #1609). Kansas State University is accredited by the Association for Assessment and Accreditation of Laboratory Animal Care International (AAALAC). Mice were housed humanely according to university, state, and federal guidelines in the AAALAC-accredited animal resource facilities of the Kansas State University College of Veterinary Medicine. The mice were housed in a clean facility with controlled humidity and temperature on 12-hour light–dark cycles. The temperature of the room was set at 20–26 °C and the relative humidity was 30–70%. Before treatment, all the mice were acclimatized in the facility for a week. The condition of each mouse was observed every day and their body weights were monitored every other day.

## Results and discussion

### Sequence optimization

**Fig. 4:**
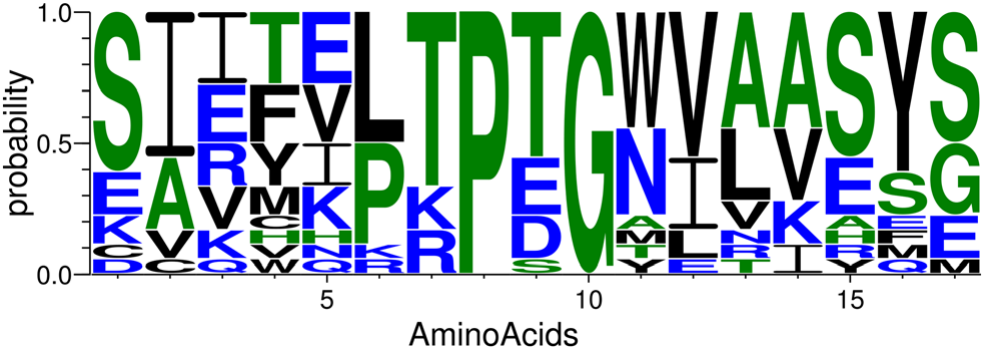
The plot of the occurrence of amino acids at specific positions in the designed peptides generated by WebLogo.^74^

We began with the X-ray structure of the complex CTLA4:B7-2, excising a 17-residue peptide (residues 85 to 101 inclusive) from B7-2. This template peptide, with the sequence CIIHHKKPTGMIRIHQM, consists of a β-hairpin with a PTG turn (residues 8 to 10). As discussed further below, the final configuration of the complex bore little resemblance to this template, so another β-hairpin template could probably have been used with similar results. Many structural and sequence variations of the template were created by flexible docking^43^ of the template peptide and sequence optimization^44^ of the resulting backbone poses. The sequence logo in the Fig. 4 represents the probability of the amino acids found at the individual position in the candidate peptides.^74^ Due to the particular geometry of the turn, the docking and optimization procedure maintained a PXG sequence of the turn, where X is usually Thr, Glu, Asp, or Ser. The amino acids around the turn were the most consistent, with many peptides adopting the subsequence LTPTGWV near the turn, although this region of the template had the subsequence KKPTGMI.

### Screening of peptide sequences

To quickly screen the peptide complex structures generated by Rosetta,^43,44^ we performed molecular dynamics simulations of these complexes in explicit solvent. Before these simulations, we cyclized the peptides using a head-to-tail amide linkage, which can improve proteolytic and conformational stability.^23–28^ We characterized the strength of binding by tracking the time that the peptide stayed bound during the simulation and estimated binding free energy using the MM-GBSA method.^49^ While neither of these characterizations are rigorous, they provided an efficient means to eliminate unpromising candidates with a small investment of computer time. For instance, the time remaining bound is stochastic and would vary among different realizations of the simulation (with different random forces from the Langevin thermostat). However, peptides with very short binding times (<100 ns) are unlikely to exhibit a high affinity for CTLA4 and can be quickly eliminated. Moreover, while the MM-GBSA (or the similar MM-PBSA) method is typically less accurate than rigorous free energy calculations,^36^ we used it for initial screening of the complex structures generated by Rosetta because of its much lower computational cost.

**Fig. 5:**
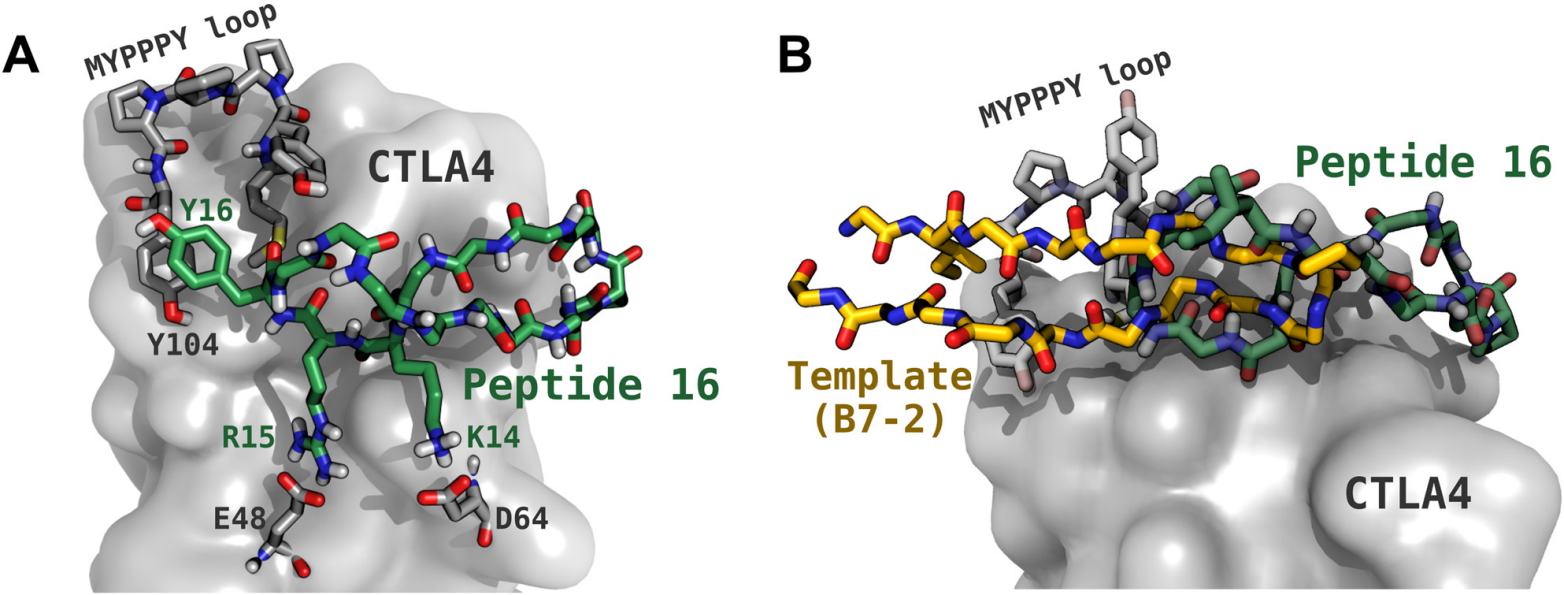
Structure of the complex between peptide 16 and CTLA4. (A) The peptide backbone is shown with green carbon atoms, with the key side chains shown (Pro8, Lys14, Arg15, Tyr16). CTLA4 is shown as a gray surface, with some key residues shown explicitly, including the MYPPPY loop (residues 99 to 104), Glu48, and Asp64. Residue numbering for CTLA4 follows that used in PDB ID 1I85. (B) Comparison of the binding configurations of peptide 16 and the fragment of B7-2 (CD86) that was used as its template.

**Fig. 6:**
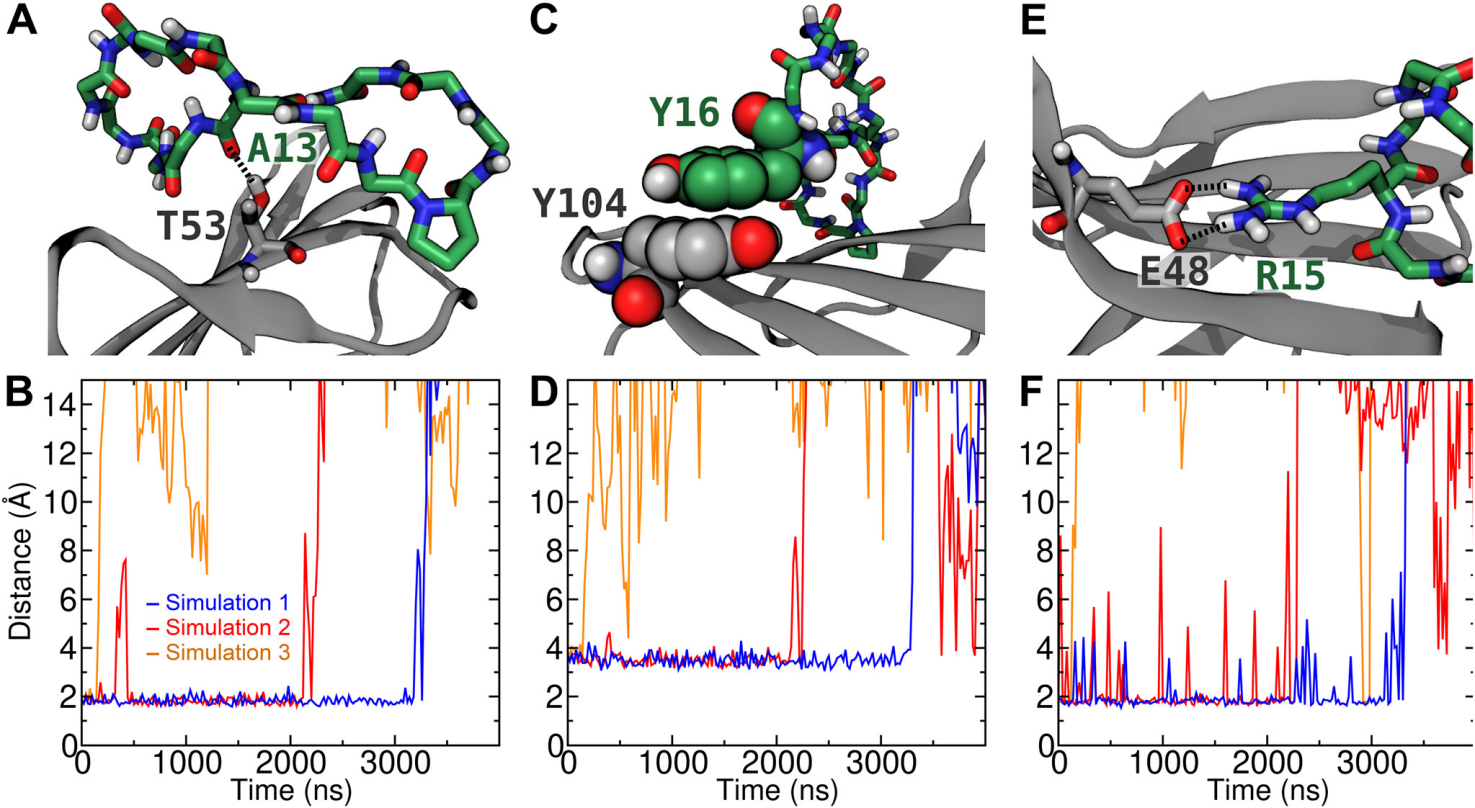
Stable contacts between peptide 16 and CTLA4 in the bound complex. (A) Simulation snapshot showing a hydrogen bond between the backbone carbonyl oxygen of Ala13 of the peptide and the side chain alcohol group of Thr53 of CTLA4. (B) Distance between this carbonyl oxygen and the alcohol hydrogen in 3 different unbiased simulations. (C) Simulation snapshot showing a π–π stacking interaction between Tyr16 of the peptide and Tyr104 of CTLA4. (D) Minimum distance between any of the phenyl carbon atoms of Tyr16 and any of the same atoms in Tyr104. (E) Simulation snapshot showing a salt-bridge between Arg15 of the peptide and Glu48 of CTLA4. (F) Minimum distance between any of the side chain NH hydrogens of Arg15 and any of the two carboxylate oxygens of the side chain of Glu48 during the 3 simulations.

We find moderate correlation (*r* = 0.59) between the time bound and the MM-GBSA free energy, which supports the usefulness of these measures. The MM-GBSA calculation is performed only over the bound portion of the simulation, so the unbinding should not bias Δ*G*_MM-GBSA_. In particular, the 4 peptides with the weakest MM-GBSA energies (Δ*G*_MM-GBSA_ > −12 kcal mol^−1^) had the shortest bound durations (<50 ns). Table 1 shows that a cyclized version of the original template (peptides T0, T1, and T2) performed rather poorly compared to the some of the generated sequences. Peptide 12 (cyc-SIITVLTPTGWVAAEFS) had the most favorable Δ*G*_MM-GBSA_ (−32.8 ± 0.1 kcal mol^−1^) and the third longest bound duration (1100 ns) of the 17 different peptides. On the other hand, while peptide 16 (cyc-EIDTVLTPTGWVAKRYS) had only a moderately favorable Δ*G*_MM-GBSA_ of −22.3 ± 0.1 kcal mol^−1^, it was never observed to dissociate within the maximum duration of these simulations (2000 ns). The structure of the complex of peptide 16 with CTLA4 is shown in Fig. 5A. For this peptide, we also performed replicate simulations beginning from a similar conformation and observed dissociation at 3300, 2300 and 200 ns (see Fig. 6).

### Rigorous calculation of binding free energy

We selected the most promising candidates in the screening calculations for rigorous free energy calculation using a variation of the BFEE method.^36,52^ We chose the peptide with the lowest MM-GBSA energy (peptide 12) and the peptide with the longest time bound (peptide 16). Both of these peptides exhibited favorable binding to CTLA4, with standard binding free energies of −10.2 ± 2.4 and −6.6 ± 3.5 kcal mol^−1^, respectively. Hence, as expected from the MM-GBSA estimate, peptide 12 showed a stronger affinity than peptide 16, although the rigorous method predicted weaker binding than MM-GBSA in both cases (Table 1). It should be noted that binding of the peptides to CTLA4 appears to be by an induced fit mechanism, as the free energies for applying the RMSD restraints to the peptide in solution (stage 1) are quite unfavorable. Similarly, Fig. S1A of the ESI† shows that peptide 16 exhibits little propensity to adopt conformations similar to the initial β-hairpin in solution, while conformations <1.5 Å in RMSD from the β-hairpin structure are favored when the peptide is bound to CTLA4 (Fig. S1H†).

The BFEE method makes these calculations feasible by first calculating the free energy to apply conformational and orientational restraints to the free ligand, then calculating the binding free energy under these restraints, and finally calculating free energy for releasing the restraints on the protein-bound ligand. Due to the path independence of free energy, the result is in principle equal to the unbiased free energy for association in absence of any restraints. The free energies for each stage of the calculation for peptide 16 are shown in Table 2. A similar table for peptide 12 can be found in Table S2 of the ESI.† The largest magnitude contributions for both peptides are for binding of the restrained ligand (stage 3), which requires the greatest amount of simulation time for convergence, followed by applying and releasing the conformational restraints (stages 1 and 2). The relative importance of these stages is consistent with previous applications of this method to peptide–protein complexes.^36^

The rigorous free energy calculations predicted a greater affinity for peptide 12 than peptide 16. However, two different commercial custom peptide services were unable to synthesize peptide 12, likely due to its low solubility. While peptide 12 has only one charged amino acid (Glu15) and six strongly hydrophobic amino acids, peptide 16 has four charged amino acids (Glu1, Asp3, Lys14, Arg15) and one fewer strongly hydrophobic amino acid, conferring much greater solubility. Hence, synthesis was successful for peptide 16 and it was chosen for our further studies. The structure of the bound complex is shown in Fig. 5.

### Structure of the complex between peptide 16 and CTLA4

As can be seen in Fig. 5A, we predict that peptide 16 adopts a twisted β-hairpin conformation and occludes a portion of the conserved MYPPPY loop, which is associated with binding its natural ligands B7-1 (CD80) and B7-2 (CD86).^15–17^ During the docking step and subsequent MD simulation, the peptides sometimes strayed far from the original binding configuration of the template. This was true for peptide 16, whose predicted stable binding configuration coincides little with that of the template fragment of B7-2 (Fig. 5B). Notably, in peptide 16, the PTG turn common to both peptides has been displaced about 15 Å farther from the MYPPPY loop. Furthermore, the orientation of peptide 16 is flipped with respect to the template peptide: for example, residue I86 of B7-2 is oriented toward CTLA4, while the corresponding residue in peptide 16, I2, is oriented toward the solution. As shown in Fig. S3A of the ESI,† the cyclized template peptide with its original coordinates did not remain bound for a long time (<100 ns) to CTLA4 in the absence of the rest of the B7-2 protein. Hence, it seems that choosing a template from B7-2 provided no benefit and equally good or better results might have been obtained with another template. In any case, the presence of peptide 16 in the configuration shown in Fig. 5B appears to preclude binding of residues 85 to 101 of B7-2 and, therefore, peptide 16 might serve as a checkpoint inhibitor.

### Intermolecular interactions in the complex

To further verify the stability of the complex of peptide 16 with CTLA4 and identify stable interactions, we performed three unbiased simulations at 310 K beginning from the complex structure predicted to have the lowest free energy by the MM-GBSA method, which was also the structure used as the reference in the rigorous free energy calculation (Fig. 5). The plots in Fig. 6 are distance plots for the most stable contacts between peptide 16 and CTLA4. As can be discerned from these plots, the complex remained bound for different lengths of time in the three replicate simulations. In simulation 3, the peptide dissociated from the binding site after a relatively short period of time (<200 ns), while in the other two simulations it remained bound for multiple microseconds.

Fig. 6A depicts a particularly stable hydrogen bond between the backbone of Ala13 of peptide 16 and Thr53 of CTLA4. In simulation 1, this H-bond remained quite stable for 3180 ns, with an average O⋯H distance of 1.8 Å (Fig. 6B). After *t* = 3180 ns, the complex dissociated, rupturing this H-bond and other peptide–protein contacts. Interestingly, in simulation 2, the H-bond between Ala13 and Thr53 broke at *t* = 356 ns before reforming at *t* = 431 ns, while the other specific interactions remained intact. These other specific interactions included a stable π–π stacking interaction between Tyr16 of the peptide and Tyr104 of CTLA4 (Fig. 6C), which is the final residue of the conserved MYPPPY loop. As shown in Fig. 6D, the minimum distance between atoms of the phenyl groups of these residues is on average 3.5 Å while peptide 16 remains bound to the binding site. A salt bridge between Arg15 of the peptide and Glu48 of CTLA4 (Fig. 6E) also appears to stabilize the bound state of the complex. As shown in Fig. 6F, this contact ruptured intermittently, although it was present (minimum distance <2.2 Å) 91% of the time while the complex was bound. Another salt bridge between Lys14 of the peptide and Asp64 was present less often (41% of the time), as illustrated in Fig. 5A and quantified in Fig. S4 of the ESI.†

### Binding assay by bio-layer interferometry

**Fig. 7:**
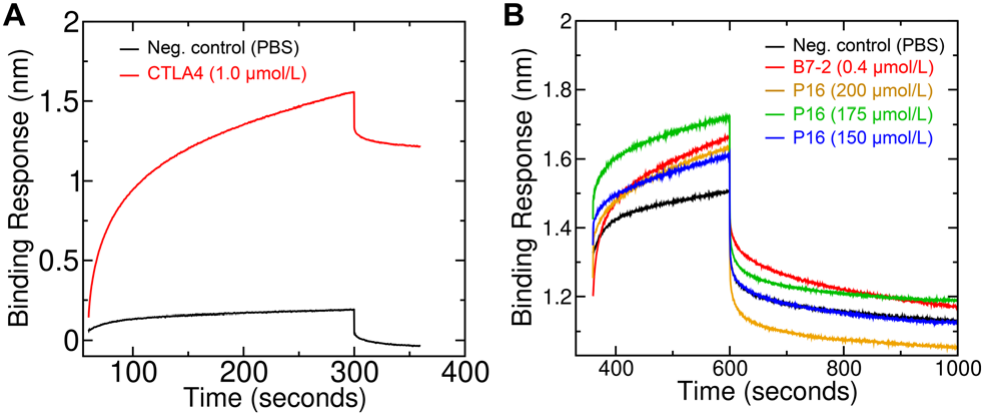
Bio-layer interferometry measurements of the binding of the designed peptide (peptide 16) to CTLA4. (A) Loading phase of the bio-layer interferometry experiment, showing that biotinylated CTLA4 is loaded on the biosensor tip. (B) Association (beginning at 360 s) and dissociation phases (beginning at 600 s) of the bio-layer interferometry experiment. B7-2, a natural binding partner of CTLA4, was included as a positive control. Three different concentrations of peptide 16 (“P16”) were used.

A bio-layer interferometry-based binding assay was performed to determine the binding affinity of peptide 16 for the CTLA4 protein. First, a PBS solution of biotinylated recombinant human CTLA4 Fc-conjugate was applied to the streptavidin coated biosensor, showing clear signs of immobilization (Fig. 7A). For comparison, a much weaker signal was seen when applying only PBS at the loading step. To further verify the proper loading of CTLA4 onto the biosensor, we performed a positive control experiment with human B7-2, one of CTLA4's natural binding partners. Analysis of the binding kinetics gave a dissociation constant (*K*_D_) of 61 ± 4 nmol L^−1^, which is roughly similar to previous reports for the human CTLA4 to B7-2 interaction.^75^ The experiments were then performed with three different concentrations of peptide 16 (150, 175, and 200 μmol L^−1^). The associated binding kinetics are shown in Fig. 7B. The resulting *K*_D_ value for the designed peptide 16 was estimated to be 31 ± 9 μmol L^−1^, which agrees well with the 15 μmol L^−1^ predicted by rigorous simulation-based method. However, given the large uncertainty of this calculated standard binding free energy (±3.5 kcal mol^−1^), any value between 0.04 and 5200 μmol L^−1^ could be considered to be in agreement.

### Peptide treatment increased cytotoxicity of T cells to cancer cells

To evaluate the effect of the newly designed CTLA4-binding peptide (denoted peptide 16 or P16) on the ability of immune cells to kill cancer cells, we co-cultured Lewis lung carcinoma (LLC) cells with antigen-primed CD8^+^ T cells (AP-CD8^+^ T cells) under different treatment regimens. The antigen-specific cytotoxic CD8^+^ T cells were generated by *in vivo* stimulation. We co-cultured immature dendritic cells (JAWSII) with irradiated Lewis lung carcinoma (LLC) cells, injected the mixture into mice, and isolated the resulting AP-CD8^+^ T cells from their spleens. The cytoxicity of these AP-CD8^+^ T cells toward LLC cells in the presence and absence of the designed peptide was then evaluated in an *in vitro* co-culture system using flow cytometry.

**Fig. 8:**
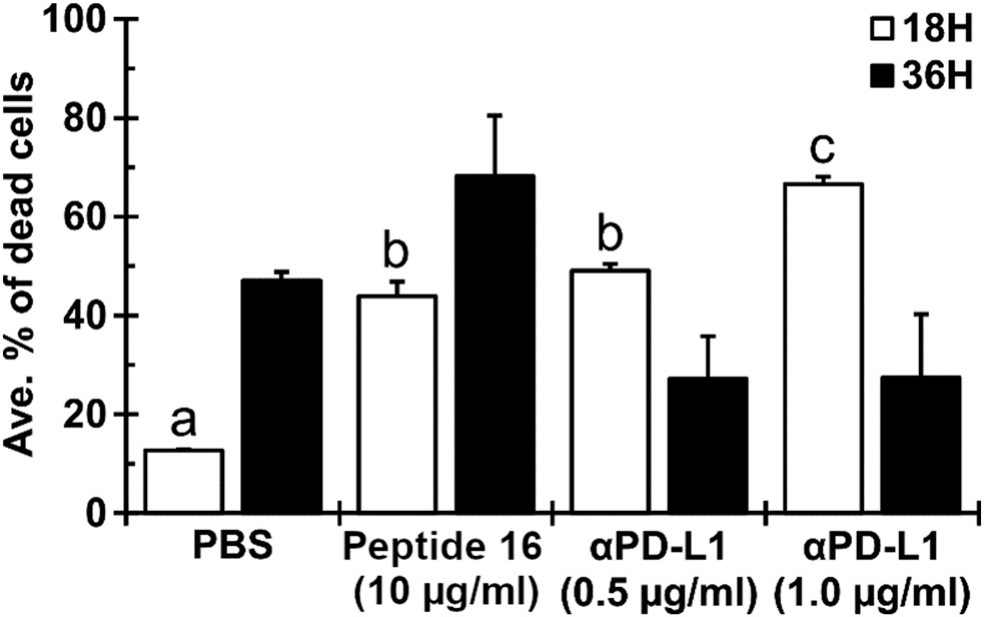
The designed peptide (peptide 16) enhances cytotoxicity of antigen-primed CD8^+^ T cells toward Lewis lung carcinoma (LLC) cells. LLC cells were co-cultured with antigen-primed CD8^+^ T cells with a 1:16 ratio. The cells were treated with peptide 16 (10 μmol L^−1^) or anti-PD-L1 antibody (αPD-L1; 0.5 or 1.0 μg mL^−1^) immediately after co-culturing. After 18 and 36 hours of co-culturing, dead cells were identified by flow cytometry. Results are presented as mean ± SD (*n* = 2). The labels a, b, and c indicate statistical significance of *p* < 0.05 between different characters.

As shown in Fig. 8, the P16 treatment appeared to induce the AP-CD8^+^ T cells to kill a greater proportion of the LLC cells than the negative control treatment (PBS). The ratio of dead LLC cells to total LLC cells under treatment with 10 μmol L^−1^ of P16 was (43.8 ± 3.0)% (mean ± SD), significantly larger (*p* < 0.05) than that under treatment with the negative control, (12.7 ± 0.3)%. Treatment with the mouse anti-PD-L1 antibody (αPD-L1, 1.0 μg ml^−1^), which was used as a positive control for immune checkpoint inhibition, showed the highest efficacy in promoting LLC cell death, resulting in (66.6 ± 1.5)% of the LLC cells being dead, significantly greater (*p* < 0.05) than all other groups. However, the effect of P16 was similar to that of 0.5 μg ml^−1^ αPD-L1: (49.1 ± 1.4)%, which was also significantly greater than that of the PBS control. The effect of P16 was sustained up to the 36 hour time point ((68.2 ± 12.3)%, not significant) after co-culture, while that of both 0.5 and 1.0 μg ml^−1^ αPD-L1 appeared to be diminished by 36 hours, to absolute values of (27.2 ± 8.7)% and (27.5 ± 12.9)%, respectively. These results suggest that the designed peptide (peptide 16) effectively inhibits binding between CTLA4, which is expressed on AP-CD8^+^ T cells, and B7-1/B7-2 (CD80/CD86), expressed by LLC cancer cells, promoting the cytotoxic effect of AP-CD8^+^ T cells.

### Peptide treatment reduced growth of tumors in mouse lungs

**Fig. 9:**
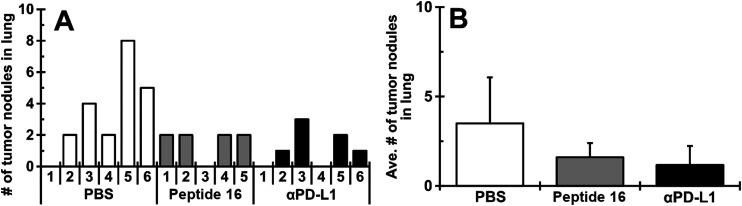
Peptide 16 treatment attenuates growth of LLC tumors in mouse lungs. All mice were inoculated with LLC cells and, 5 days later, intravenously injected with JAWSII cells stimulated by co-culturing with irradiated LLC (JAWS-irrLLC). Mice were randomly divided into 3 groups and intraperitoneally treated with PBS, CTLA4-binding peptide (peptide 16, 10 mg kg^−1^ per day) and anti-PD-L1 antibody (αPD-L1, 10 mg kg^−1^ per day). The number of tumor nodules and their sizes were recorded at the end of the experiment. The number of tumor nodules in each mouse (A) and the average number in each group (B). Averages are presented as mean ± SD (*n* = 5–6).

The effect of the designed peptide (P16) on tumor growth in the lung was evaluated using an LLC mouse allograft model. To enhance antitumor immunity of the host, a mouse dendritic cell line (JAWSII) was co-cultured with irradiated LLC cells for 24 hours (denoted JAWS-irrLLC) and injected into all mice at 5 days after intravenous inoculation of LLC cells. The treatment with the CTLA4-binding peptide (P16, 10 mg kg^−1^ per day) or anti-PD-L1 antibody (αPD-L1, 10 mg kg^−1^ per day), the positive control, was started from 2 days after JAWS-irrLLC injection. As shown in Fig. 9A and B, in macroscopic observations, a smaller number of tumor nodules was confirmed for the group treated with P16 (4 out of 5, mean ± SD, 1.60 ± 0.80) compared to the negative control group, treated with JAWS-irrLLC only (5 out of 6, 3.50 ± 2.57). The result with peptide 16 was similar to that with the positive control, αPD-L1, (4 out of 6, 1.17 ± 1.07). Similar trends were seen in the total tumor volume. These results suggest that novel designed peptide P16 promotes anticancer immunity, attenuating tumor growth.

We considered the possibility that this anticancer immunity might be due to alteration of cellular immunity-related lymphocyte populations in the treated groups. However, as shown in Fig. S5 of the ESI,† no significant trends emerged.

## Conclusions

In this contribution, we used a protein-modeling program (Rosetta) and molecular dynamics simulation to design a cyclic peptide that binds to CTLA4. The resulting peptide, cyc(EIDTVLTPTGWVAKRYS), denoted peptide 16, was demonstrated experimentally to bind CTLA4 by a physical method (bio-layer interferometry). Our cell-culture studies suggested that peptide 16 promoted the cytotoxic effect of antigen-primed CD8^+^ T cells on co-cultured cancer cells. In mice, we found that peptide 16 significantly reduced tumor growth in a lung cancer allograft model.

While creating a template from the experimental structure of the CTLA4:B7-2 complex seemed like a good starting point, the bound configuration of peptide 16 bears little resemblance to this template. Hence, choosing this template likely conferred no particular advantage. Therefore, we could have perhaps chosen a β-hairpin template known to be more conformationally stable. Despite this, we believe that the rest of the peptide design workflow presented here could be useful in development of other therapeutic peptides.

The dissociation constant measured experimentally, 31 ± 4 μmol L^−1^, is not as strong as existing monoclonal antibodies; however, peptide 16, a 17-residue cyclic peptide, likely could be manufactured more economically. Moreover, there is potential to further optimize peptide 16. The rigorous free energy calculation revealed that the β-hairpin structure that the peptide adopts when bound is unfavorable in solution, leading to a significant free energy cost for binding associated with an unfavorable conformational entropy change (stage 1 in Table 2 and Fig. S1†). Artificial amino acids or synthetic linkages might be used to better stabilize the β-hairpin structure, giving a more favorable Δ*G*^apply^_RMSD_, without disrupting other favorable contributions to the interaction with CTLA4. With such potential improvements, it seems likely that such designed peptides could serve as immunomodulators for cancer immunotherapy and other immunotherapy applications.

## Data and software availability

Data for molecular dynamics simulations of the designed cyclic peptide (peptide 16) bound to CTLA4 are freely available for download from Zenodo (https://doi.org/10.5281/zenodo.7186684). The archive includes all files needed to run and analyze simulations like those described detailed in Fig. 6. They include molecular model structure files (in CHARMM/NAMD psf), force field parameter files (in CHARMM format), initial atomic coordinates (pdb format), NAMD configuration files, NAMD log files, and NAMD output including restart files (in binary NAMD format) and trajectories in dcd format (downsampled to 10 ns per frame). Analysis is controlled by a shell script (Bash-compatible) that calls VMD Tcl scripts. These scripts and their output are also included.

## Author contributions

Ravindra Thakkar: investigation, methodology, visualization, writing – original draft, writing – review & editing. Deepa Upreti: investigation, writing – review & editing. Susumu Ishiguro: investigation, methodology, visualization, writing – review & editing. Masaaki Tamura: conceptualization, methodology, funding acquisition, supervision, resources, writing – review & editing. Jeffrey Comer: conceptualization, investigation, methodology, software, funding acquisition, supervision, visualization, writing – review & editing.

## Conflicts of interest

RT, MT, and JC have a pending patent covering the peptide designed in this work (peptide 16).

## Supplementary Material

MD-014-D2MD00409G-s001
