# Supplementary material for: Computational design of a cyclic peptide that inhibits the CTLA4 immune checkpoint
Source: RSC Med Chem. 2023 Mar 1;14(4):658–70. doi: 10.1039/d2md00409g (PMC10131585; doi:10.1039/d2md00409g)
Supplement: MD-014-D2MD00409G-s001 [file MD-014-D2MD00409G-s001.pdf]

Electronic Supplementary Material (ESI) for RSC Medicinal Chemistry.
This journal is © The Royal Society of Chemistry 2023

*Electronic Supplementary Information*

# Computational design of a cyclic peptide that inhibits the CTLA4 immune checkpoint

Ravindra Thakkar
Department of Anatomy and Physiology, Kansas State University, Manhattan, Kansas, 66506-5802

Deepa Upreti
Department of Anatomy and Physiology, Kansas State University, Manhattan, Kansas, 66506-5802

Susumu Ishiguro
Department of Anatomy and Physiology, Kansas State University, Manhattan, Kansas, 66506-5802

Masaaki Tamura
Department of Anatomy and Physiology, Kansas State University, Manhattan, Kansas, 66506-5802

Jeffrey Comer
Department of Anatomy and Physiology, Kansas State University, Manhattan, Kansas, 66506-5802
E-mail: jeffcomer@ksu.edu

| ID | Sequence | Rosetta Score | Time Bound (ns) | $\Delta G_{\text{MM-GBSA}}$ (kcal/mol) |
|---|---|---|---|---|
| 1 | `cyc(CIVHENRPEGLVRVHLC)` | 167.9 | 23.6 | $-5.16 \pm 0.30$ |
| 2 | `cyc(CVVFEPKPEGTEKVHEC)` | 211.6 | 74.2 | $-19.60 \pm 0.53$ |
| 3 | `cyc(CIIWEDQPNGKVCVHSC)` | 165.2 | 89.6 | $-10.99 \pm 0.28$ |
| 4 | `cyc(CVIEQYRPEGVVLIYEC)` | 172.2 | 578.8 | $-18.17 \pm 0.14$ |
| 5 | `cyc(CTVAIPLPDGKICVKSC)` | 161.0 | 59.6 | $-12.97 \pm 0.32$ |
| 6 | `cyc(CEVRKYSESGVIPIDSC)` | 176.3 | 106.0 | $-8.11 \pm 0.27$ |
| 7 | `cyc(CLITAASESGVYTIYEC)` | 169.4 | 4.6 | $-3.95 \pm 1.03$ |
| 8 | `cyc(CVLQQNAPEGIITIEEC)` | 161.9 | 28.0 | $-15.51 \pm 0.94$ |
| 9 | `cyc(CQITVPLPEGVVIVETC)` | 165.0 | 13.8 | $-5.66 \pm 0.65$ |
| 10 | `cyc(CLIVEYKPEGVEIIYEC)` | 191.8 | 37.6 | $-7.90 \pm 0.34$ |
| 11 | `cyc(CSTKQNMPEGTVLIYSC)` | 181.6 | 5.8 | $-2.29 \pm 0.85$ |
| 12 | `cyc(CVVSQDRPEGTVLLYTC)` | 164.2 | 402.4 | $-13.20 \pm 0.15$ |
| 13 | `cyc(CVIAYAAPEGYILVTVC)` | 223.4 | 162.2 | $-19.03 \pm 0.19$ |

**Table S1:** Evaluation of the binding affinity for disulfide-cyclized candidate peptides. For each sequence, we include the Rosetta score (lower is more favorable), the time that the peptide remained bound to CTLA4 in an MD simulation, and the binding free energy as estimated by the MM-GBSA method.

| Stage | System | Action | Free-Energy Term | Free Energy (kcal/mol) | Time (ns) |
|---|---|---|---|---|---|
| 1 | ligand | Apply conform. restraint | $\Delta G_{\text{RMSD}}^{\text{unbound}}$ | $+9.48 \pm 1.73$ | 381 |
| 2† | ligand | Apply orient. restraints | $\Delta G_{\Theta\Phi\Psi}^{\text{unbound}}$ | $+6.80 \pm 0.00$ | 0 |
| 3 | complex | Binding of restrained ligand | $-k_{\text{B}}T\ln(S^*I^*C^\circ)$ | $-13.48 \pm 0.74$ | 1582 |
| 4 | complex | Release $\phi$ direct. restraint | $\Delta G_\phi$ | $-0.29 \pm 0.08$ | 19 |
| 5 | complex | Release $\theta$ direct. restraint | $\Delta G_\theta$ | $-0.08 \pm 0.01$ | 20 |
| 6 | complex | Release $\Psi$ orient. restraint | $\Delta G_\Psi$ | $-0.50 \pm 0.24$ | 18 |
| 7 | complex | Release $\Phi$ orient. restraint | $\Delta G_\Phi$ | $-0.55 \pm 0.27$ | 17 |
| 8 | complex | Release $\Theta$ orient. restraint | $\Delta G_\Theta$ | $-0.23 \pm 0.05$ | 18 |
| 9 | complex | Release conform. restraint | $\Delta G_{\text{RMSD}}$ | $-11.35 \pm 1.47$ | 99 |
| Total | – | Sum | $\Delta G^\circ$ | $-10.21 \pm 2.41$ | 2154 |

**Table S2:** Free-energy values and simulation times for each stage of the rigorous calculation of the absolute free energy for binding of Peptide 12 to CTLA4. Note that the numbering of the stages is reversed to compared that presented in Fu et al.[3] First, we calculate free energies required to apply conformational (stage 1) and orientational restraints (stage 2) to the free peptide. Next, the binding free energy for the complex is calculated with these restraints applied to the peptide, along with directional restraints for the position of the peptide relative to the protein. Finally, we calculate the free energy of releasing these directional, orientational, and conformational restraints. Stage 2 (†) was computed analytically and requires no simulation. Stage 3 was calculated using replica-exchange umbrella sampling. All remaining stages were computed with ABF.

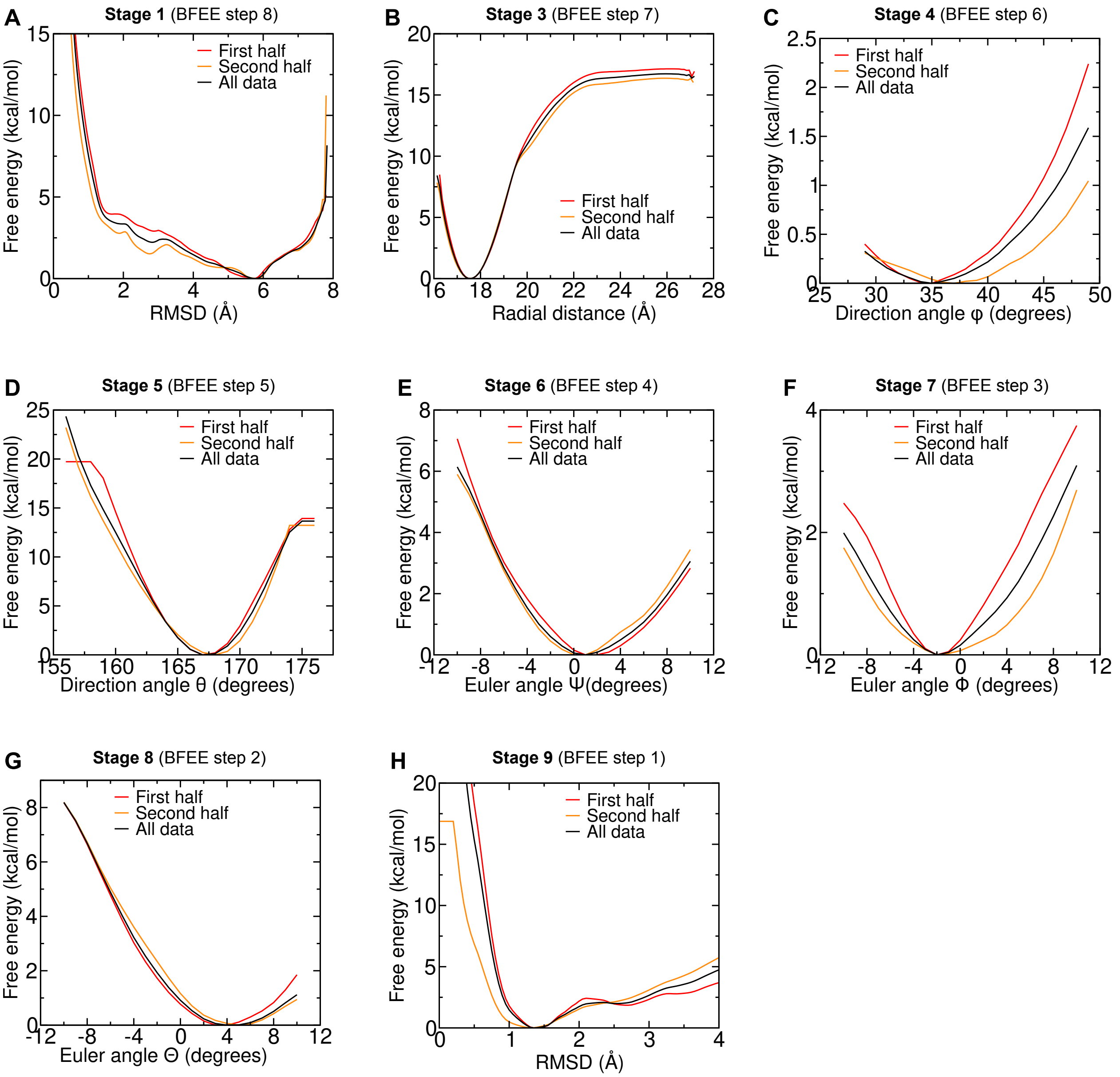


**Figure S1:** Convergence of rigorous free energy calculations for Peptide 16. For each stage of the calculation, we plot the potentials of mean force (PMFs) obtained from the first half of the data and the second half of the data. For stages 1 and 4–9, we calculate the gradient in each ABF bin for only the second half by $g_i^{(2)} = (n_i^{\mathrm{F}} g_i^{\mathrm{F}} - n_i^{(1)} g_i^{(1)})/(n_i^{\mathrm{F}} - n_i^{(1)})$, where $n_i^{(1)}$ and $g_i^{(1)}$ is the count and mean gradient for bin $i$ halfway through the simulation and $n_i^{\mathrm{F}}$ and $g_i^{\mathrm{F}}$ is the count and mean gradient for bin $i$ at the end of the simulation.[1] For stage 3, the PMFs were calculated by umbrella sampling rather than ABF, so the first- and second-half PMFs were computed simply by only feeding data from the appropriate halves into the weighted histogram anaysis method.[2] The stages are numbered as in Table S2, as well as with the numbering convention of the BFEE plugin. The quality of the convergence can be estimated by comparing the PMFs from the first and second halves.

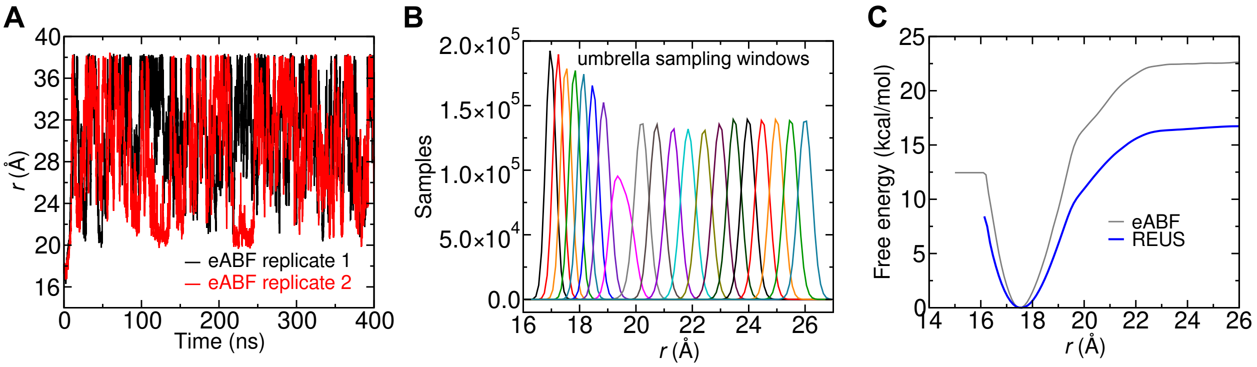


**Figure S2:** Comparison of extended adaptive biasing force (eABF)[4] and replica-exchange umbrella sampling (REUS)[5] for calculating the potential of mean force along the radial coordinate $r$ under conformational and orientational restraints[3,6] on the ligand (Peptide 16). **(A)** The value of the radial coordinate $r$ as a function of time in the eABF calculations for two replicates. In both cases, the peptide leaves the bound state ($r < 19$ Å) early in the simulation and never returns. **(B)** Sampling of the transition coordinate $r$ in each of the 20 REUS windows. The histograms are sampled on 0.1 Å bins. Overall sampling for REUS is much more uniform than for eABF and configurations cross between the bound and unbound states multiple times instead of only once. **(C)** The potentials of mean force along $r$ calculated by the eABF and REUS. The results are significantly different owing to the poor sampling for $r < 19$ Å in the eABF calculation.

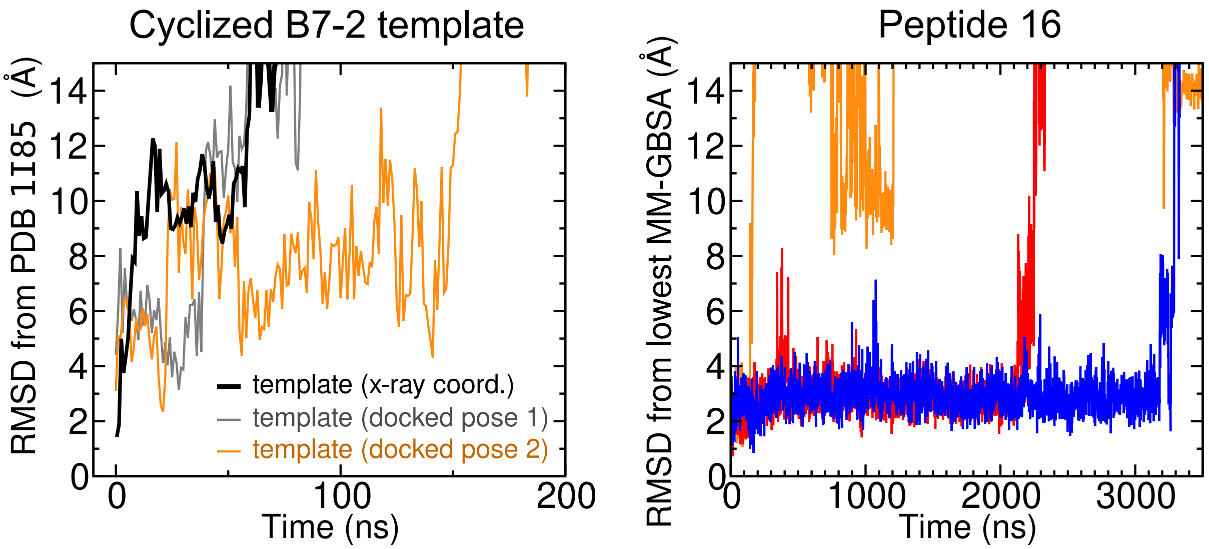


**Figure S3:** RMSD for a cyclized template and Peptide 16 in molecular dynamics simulations. In all cases, the simulation frame is fit to the reference structure by a rigid transformation to superimpose the binding site of CTLA4 (VMD selection “sequence MYPPPY and name CA”) in the two structures. The RMSD is then calculated from the difference in the position of peptide $C_\alpha$ carbons between the simulation frame and the reference without any further transformations. (A) RMSD from the original x-ray coordinates (PDB ID: 1I85) for a cyclic peptide made from residues 85–101 of B7-2. The black curve shows the RMSD for the original 1I85 coordinates (Peptide T0). Because the peptide rapidly dissociated with these initial coordinates (in $<$10 ns), we tried redocking the peptide to produce two slightly different poses (RMSD values of 3.3 and 3.9 Å from the original coordinates). Dissociation was also quite rapid with these poses. (B) RMSD from the coordinates for the lowest MM-GBSA energy for Peptide 16 in three simulations with different initial conditions.

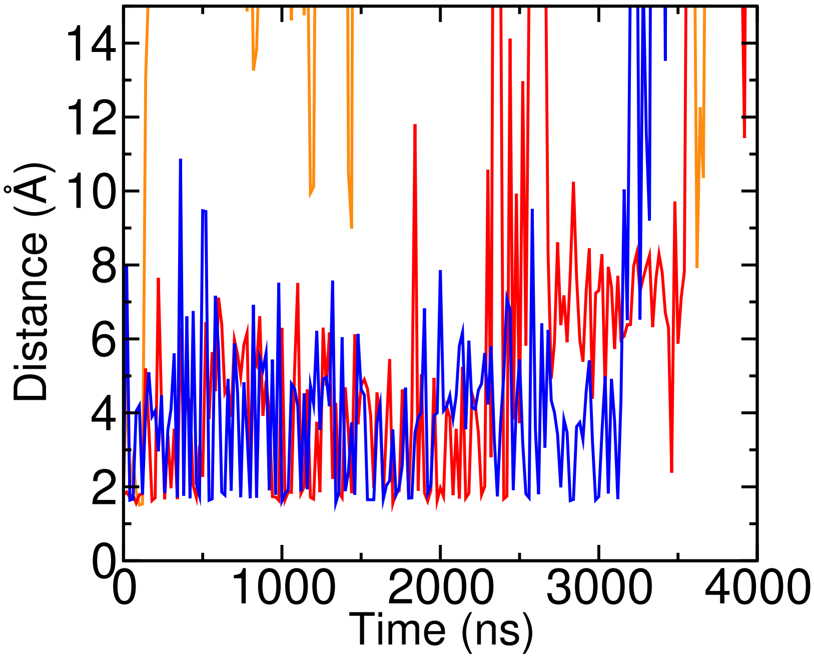


**Figure S4:** Minimum distance between any of hydrogens of the $NH^{3+}$ group of Lys14 of the peptide and any of the two carboxylate oxygens of the side chain of Asp64 of CTLA4 during the 3 simulations.

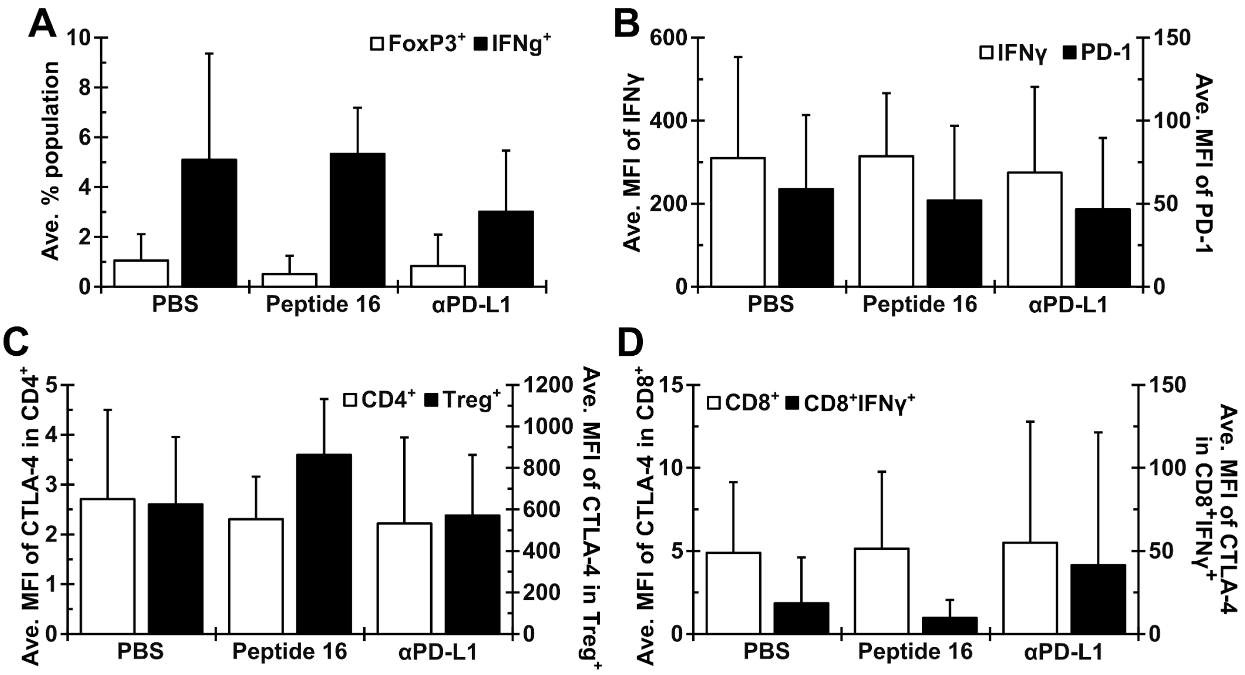


**Figure S5:** No clear differences were seen in cell populations among the treatment groups. Plotted are the percent population of CD4$^+$ FoxP3$^+$ regulatory T cells and CD8$^+$ IFN$\gamma$+ cytotoxic T cells, and the expression of immune check point molecules in mouse blood. (A) Average percent population of CD4$^+$ FoxP3$^+$ regulatory T cells (Treg) and CD8$^+$ IFN$\gamma$+ cytotoxic T cell (IFN$\gamma$). (B) Average mean fluorescence intensity (MFI) of IFN$\gamma$ and PD-1 in CD8$^+$ cytotoxic T cells. (C) Average MFI of CTLA4 in CD4$^+$ T cells (open bar) and Treg (filled bar). (D) Average MFI of CTLA4 in CD8$^+$ cytotoxic T cell (open bar) and CD8$^+$ IFN$\gamma$+ activated cytotoxic T cell (filled bar). All mice were injected with mouse dendritic cells (JAWSII) co-cultured with with irradiated LLC cells to enhance antitumor immunity of the host. "JAWS-irrLLC alone" refers to the group of these mice afterward treated only with PBS, the negative control. "CTLA-4ip" refers to the group treated with the designed peptide denoted Peptide 16. "$\alpha$PD-L1" refers to the group of mice treated with the positive control, a anti-PD-L1 antibody.

## References


1. Poblete, H.; Miranda-Carvajal, I.; Comer, J. Determinants of Alanine Dipeptide Conformational Equilibria on Graphene and Hydroxylated Derivatives. *J. Phys. Chem. B* **2017**, *121*, 3895–3907.
2. Roux, B. The Calculation of the Potential of Mean Force Using Computer Simulations. *Comp. Phys. Commun.* **1995**, *91*, 275–282.
3. Fu, H.; Cai, W.; Hénin, J.; Roux, B.; Chipot, C. New Coarse Variables for the Accurate Determination of Standard Binding Free Energies. *J. Chem. Theory Comput.* **2017**, *13*, 5173–5178.
4. Comer, J.; Gumbart, J. C.; Hénin, J.; Lelièvre, T.; Pohorille, A.; Chipot, C. The Adaptive Biasing Force Method: Everything You Always Wanted to Know But Were Afraid to Ask. *J. Phys. Chem. B* **2015**, *119*, 1129–1151.
5. Murata, K.; Sugita, Y.; Okamoto, Y. Free Energy Calculations for DNA Base Stacking by Replica-exchange Umbrella Sampling. *Chem. Phys. Lett.* **2004**, *385*, 1–7.
6. Fu, H.; Gumbart, J. C.; Chen, H.; Shao, X.; Cai, W.; Chipot, C. BFEE: A User-Friendly Graphical Interface Facilitating Absolute Binding Free-Energy Calculations. *J. Chem. Inf. Model.* **2018**, *58*, 556–560.
